# Low statistical power in biomedical science: a review of three human research domains

**DOI:** 10.1098/rsos.160254

**Published:** 2017-02-01

**Authors:** Estelle Dumas-Mallet, Katherine S. Button, Thomas Boraud, Francois Gonon, Marcus R. Munafò

**Affiliations:** 1 Institute of Neurodegenerative Diseases, CNRS UMR-5293, University of Bordeaux, Bordeaux, France; 2 Centre Emile Durkheim, CNRS UMR-5116, University of Bordeaux, Bordeaux, France; 3 Department of Psychology, University of Bath, Bath, UK; 4 MRC Integrative Epidemiology Unit at the University of Bristol, Bristol, UK; 5 UK Centre for Tobacco and Alcohol Studies, School of Experimental Psychology, University of Bristol, Bristol, UK

**Keywords:** statistical power, reproducibility, neurology, psychiatry, somatic disease

## Abstract

Studies with low statistical power increase the likelihood that a statistically significant finding represents a false positive result. We conducted a review of meta-analyses of studies investigating the association of biological, environmental or cognitive parameters with neurological, psychiatric and somatic diseases, excluding treatment studies, in order to estimate the average statistical power across these domains. Taking the effect size indicated by a meta-analysis as the best estimate of the likely true effect size, and assuming a threshold for declaring statistical significance of 5%, we found that approximately 50% of studies have statistical power in the 0–10% or 11–20% range, well below the minimum of 80% that is often considered conventional. Studies with low statistical power appear to be common in the biomedical sciences, at least in the specific subject areas captured by our search strategy. However, we also observe evidence that this depends in part on research methodology, with candidate gene studies showing very low average power and studies using cognitive/behavioural measures showing high average power. This warrants further investigation.

## 1. Background

There is growing consideration of the possibility that many published research findings may be false, indicate effects that are in the opposite direction to any true effect, or indicate effects that are substantially inflated compared to any true effect [1]. All of these will increase the likelihood that research findings will prove difficult to replicate. Within the widely used null hypothesis significance testing (NHST) framework, one factor that contributes to poor reproducibility is low statistical power [2–4]. However, the relationship between statistical power and the likelihood that a statistically significant finding represents a false-positive result is under-appreciated [5]. Low statistical power (arising, for example, from low sample size of studies, small effects being investigated, or both) adversely impacts on the likelihood that a statistically significant finding actually reflects a true effect and (if the effect is indeed real) increases the likelihood that the estimate of the magnitude of that effect is inflated (also known as a type M or magnitude error) or in the opposite direction (a type S or sign error) relative to the true effect [6].

Despite the central place of statistical power within the NHST framework, there is evidence that it remains poorly understood and is often not considered at the study design stage [5,7]. A survey by Vankov *et al.* [5] of authors who had published in a leading psychology journal indicated that approximately one-third held beliefs that would, on average, serve to reduce statistical power. This included using accepted norms within their field to decide on sample size, in the belief that this would be sufficient to replicate previous results. Given evidence that many published findings report *p*-values close to the conventional 5% threshold for statistical significance [8], this belief is likely to be unwarranted. If an experiment finds an effect with a *p*-value close to 0.05, and if we assume the observed effect size is accurate, an exact replication of the experiment with the same sample size will on average reproduce that finding only 50% of the time [5]. In practice, power is likely to be lower than 50% because the effect size estimate observed in the original study will probably be an overestimate [2,9]. However, in this survey, over one-third of respondents inaccurately believed that in this scenario the finding would replicate over 80% of the time [5]. Button *et al.* [2] recently reported that studies in the neuroscience literature have a median statistical power of approximately between 8 and 31%. However, there is no particular reason to believe that the situation in the neuroscience literature is any better or worse than the situation across the biomedical sciences in general. Nevertheless, this has not been formally investigated across different domains of biomedical science to date.

We, therefore, conducted a review of meta-analyses of studies of the association of biological, environmental or cognitive/behavioural parameters with neurological, psychiatric and somatic diseases (excluding treatment studies to ensure that included studies were more directly comparable). Our intention was to estimate the average statistical power across these domains, and explore whether studies in particular domains tend, on average, to have higher or lower statistical power than in other domains. Our selection of disease domains was partly arbitrary, although we excluded, for example, stroke from neurological diseases because it is also a cardiovascular disease, and traumatic brain injury because it is accidental. Our selection of neurological diseases included four severe diseases with the highest prevalence, while our selection of four psychiatric disorders was less systematic and included two less prevalent disorders (autism and schizophrenia) and two more prevalent disorders (ADHD and unipolar depression). Finally, our selection of four somatic diseases was also largely arbitrary; these were selected because their biological causes and risk factors are still poorly understood, and because their prevalence is similar to that of the other domains.

We applied a similar methodology to that used previously by Button *et al.* [2], taking the effect size indicated by a meta-analysis as the best estimate of the likely true effect size, and estimated the average statistical power of studies in the meta-analysis to obtain *p* < 0.05 given that effect size (i.e. to reject the null hypothesis of no effect at *p* < 0.05). Whereas Button *et al.* selected meta-analyses published in a single year (2011), we selected meta-analyses from a longer period (2008–2012).

## 2. Material and methods

### 2.1. Selection of articles for inclusion

We searched PubMed for relevant articles, with the search limited to articles referenced as ‘meta-analysis’ and published in English between 1 January 2008 and 31 December 2012. Studies published online in 2012 but printed in 2013 or 2014 were also considered. We used the following key words for each disease: ‘attention deficit hyperactivity disorder’, ‘autism’, ‘major depression’, ‘epilepsy’, ‘Alzheimer's disease’, ‘Parkinson's disease’, ‘multiple sclerosis’, ‘breast cancer’, ‘glaucoma’, ‘psoriasis’ and ‘rheumatoid arthritis’.

Meta-analyses captured by this search strategy were screened by two authors (E.D.-M. and F.G.) for eligibility. First, the title and the abstract were considered. Second, full texts were obtained for the remaining studies and screened by two authors (E.D.-M. or F.G.). Articles were excluded if: (i) they were related to the treatment, screening or diagnosis of the disease, (ii) the disease itself was the risk factor for another outcome, (iii) no meta-analysis was conducted or (iv) the article was not related or relevant to the disease. Articles were included if (i) they compiled results from at least seven distinct datasets reported in at least four independent publications, (ii) the effect size was expressed as standardized mean difference (Cohen's *d*), odds ratio (OR) or risk ratio (RR) and (iii) the measured parameter was quantitative and not qualitative (e.g. self-assessed psychological trait).

Data were extracted from the main study text, tables and forest plots. A number of articles reported several meta-analyses. We included all of these providing that they dealt with distinct parameters or risk factors. If an article reported several meta-analyses on the same parameter and the same outcome, we selected the most comprehensive one (i.e. the one containing the most datasets) and the one reporting the fixed effects rather than the random effects model. We selected the result from the fixed effects model because this is less strongly influenced by small (i.e. imprecise) studies than the result from the random effects model, which may produce inflated point estimates in the presence of bias (e.g. publication bias) [10]. When two or more articles dealt with the same parameter and the same outcome, the most recent one was used. For genetic association studies, we used the meta-analysis results for the allelic model when available, as the additive model has reasonable statistical power for both additive and dominant effects. If not, we used, in order of preference and availability, the dominant, recessive or homozygote model.

### 2.2. Data extraction

Data were extracted independently by two authors (E.D.-M. and F.G.), and discrepancies resolved by mutual consent. A third author (K.S.B.) conducted a further 10% data check, which resulted in a small number of errors being detected and corrected.

The following data were extracted from the included articles: year of publication, first author, research domain (neurological, psychiatric, somatic disease), research methodology (cognitive/behavioural, genetic, brain imaging, other), number of datasets included in the meta-analysis, summary effect size estimate of the meta-analysis and its statistical significance (based on *p* < 0.05), and number of patients and controls included in the meta-analysis. When the number of patients and controls was not given, they were identified from individual studies where possible. If the effect size was reported as a mean difference, it was converted to Cohen's *d* using the software OpenMetaAnalyst [11]. Meta-analyses were excluded if data were incomplete or if the reporting was unclear.

For each meta-analysis, we calculated the average number of patients and controls (i.e. number of patients or controls included in the meta-analysis divided by the number of datasets included in the meta-analysis). In order to compare the effect sizes across all meta-analyses and diseases, effect sizes were natural logarithm transformed. The effect sizes expressed as Cohen's *d* were converted to ln(OR) using the formula ln(OR) = *d* × *π*/√3. For each disease, the average effect size and the median number of patients and controls in the contributing studies were calculated (see electronic supplementary material, table S1).

### 2.3. Statistical analysis

For each meta-analysis, we calculated an average estimate of the statistical power of individual contributing studies. We used the effect size reported in the meta-analysis as the best estimate of the likely population effect size. The validity of this approach is supported by evidence that pairs of meta-analyses published on the same topic at an interval of 5 years report very similar effect sizes [12]. Moreover, the difference between pairs of estimates is inversely correlated with the number of primary studies included in the earliest meta-analyses [12]. However, as a sensitivity analysis we repeated our analysis using only those meta-analyses that indicated a statistically significant (i.e. *p* < 0.05) pooled effect size estimate, in order to exclude cases where the true effect size may in fact have been zero, or close to zero.

The average number of patients and healthy controls was calculated by dividing the number of patients and controls included in the meta-analysis by the number of individual contributing studies. Finally, the number of events in controls was identified from the meta-analysis. Where this was not available, it was calculated from the data provided in the meta-analysis. Where the meta-analysis did not provide the number of events in controls, we identified the contributing study with the largest control sample size and extracted the number of events in controls from the corresponding original publication. The achieved statistical power of individual contributing studies to reject the null hypothesis of no effect given the effect size reported in the meta-analysis (i.e. the best estimate of the likely population effect size) was calculated using G*Power 3.1 [13], assuming an *α* level of 5%.

## 3. Results

### 3.1. Characteristics of included studies

Table 1 summarizes the number of articles selected at each step of the process (see Material and methods), and the number of meta-analyses included by disease. The selection of studies is also shown in flow diagrams (electronic supplementary material, figures S1–S12). We included 660 meta-analyses reporting a relationship between a risk factor or other parameter (e.g. biomarker) and a disease outcome in one of the three domains of interest (psychiatry, neurology, somatic disease). Among these, 68 meta-analyses focused on cognitive/behavioural measures (e.g. time off task in a classroom of children with attention deficit hyperactivity disorder), all but two of which were in the domain of psychiatry (table 1). Our search retrieved eight meta-analyses reporting studies in animals. However, as all were focused on treatment effects in animal models, they were excluded. Therefore, all meta-analyses included in the present study were related to studies in humans (see electronic supplementary material, table S2).

**Table 1.** Number of studies identified in three research domains. ADHD, attention deficit hyperactivity disorder; ASD, autism spectrum disorder; MDD, major depressive disorder; SCZ, schizophrenia; AD, Alzheimer's disease; Epi, epilepsy; MS, multiple sclerosis; PD, Parkinson's disease; BC, breast cancer; Glau, glaucoma; Pso, psoriasis; RA, rheumatoid arthritis.

	psychiatric disease				neurological disease				somatic disease			
	ADHD	ASD	MDD	SCZ	AD	Epi	MS	PD	BC	Glau	Pso	RA
PubMed search	118	71	553	454	197	147	137	139	811	75	81	285
examined full texts	43	36	114	198	117	29	44	74	345	19	22	82
included articles	20	13	29	60	35	12	18	20	87	9	8	22
included meta-analyses	40	24	54	203	50	15	37	57	110	21	15	34
cognitive/behavioural studies	14	0	9	43	1	0	0	1	0	0	0	0
biological studies	26	24	45	160	49	15	37	56	110	21	15	34

### 3.2. Statistical power

We calculated the median statistical power across the three domains of interest, separately for the individual diseases we examined (attention deficit hyperactivity disorder, autism spectrum disorder, major depressive disorder, schizophrenia, Alzheimer's disease, epilepsy, multiple sclerosis, Parkinson's disease, breast cancer, glaucoma, psoriasis, rheumatoid arthritis). On average, the median statistical power was low, ranging from less than 9% in studies of Alzheimer's disease to almost 30% in studies of major depressive disorder (table 2).

**Table 2.** Median statistical power of studies in three research domains. ADHD, attention deficit hyperactivity disorder; ASD, autism spectrum disorder; MDD, major depressive disorder; SCZ, schizophrenia; AD, Alzheimer's disease; Epi, epilepsy; MS, multiple sclerosis; PD, Parkinson's disease; BC, breast cancer; Glau, glaucoma; Pso, psoriasis; RA, rheumatoid arthritis; *k*, number of included studies.

	psychiatric disease				neurological disease				somatic disease			
	ADHD (%)	ASD (%)	MDD (%)	SCZ (%)	AD (%)	Epi (%)	MS (%)	PD (%)	BC (%)	Glau (%)	Pso (%)	RA (%)
all meta-analyses												
all studies (*k* = 660)	29.0	24.0	29.9	24.4	8.5	23.9	23.6	26.7	16.0	10.7	19.6	18.6
cognitive/behavioural (*k* = 68)	83.3	—	74.9	97.3	53.8	—	—	34.5	—	—	—	—
biological (*k* = 592)	13.1	24.0	25.3	17.5	8.2	23.9	23.6	26.0	16.0	10.7	19.6	18.6
significant meta-analyses												
all studies (*k* = 420)	51.8	77.2	38.6	49.3	35.8	64.3	61.5	45.7	43.1	67.2	22.3	26.0

Meta-analyses focused on cognitive/behavioural measures related to ADHD (number of studies *k* = 14), major depressive disorder (*k* = 9) and schizophrenia (*k* = 43) showed a much higher median statistical power, ranging from 75 to 97% (table 2). Since this type of study was seen almost exclusively in our sample of psychiatric studies (table 1), we also calculated the median statistical power excluding these studies, in order to enable a more direct comparison with neurological and somatic studies when the methodologies used were similar. The remaining meta-analyses that dealt with biological parameters indicated very similar median statistical power: 17% for somatic diseases, 20% for psychiatric disorders and 20% for neurological diseases (table 2).

**Figure 1.**
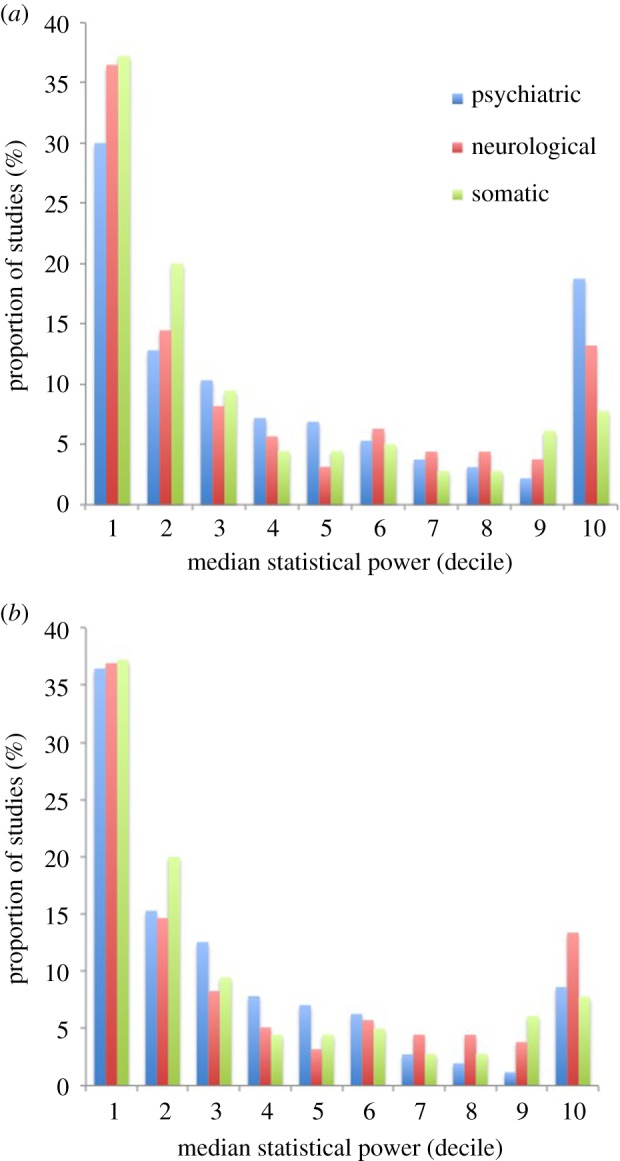
Distribution of statistical power of individual studies. The distribution of the average statistical power of individual studies contributing to meta-analyses across three biomedical domains (psychiatry, neurology and somatic disease) is shown. This clearly indicates a bimodal distribution, with the first mode indicating that the majority of studies have very low power (less than 20%), and the second mode indicating that a minority have very high power (greater than 90%). This overall pattern appears to hold across all three domains of interest and is seen in the full sample of meta-analyses (*a*) and when the subsample of meta-analyses that focus on cognitive or behavioural assessments is excluded (*b*).

We plotted the average statistical power of the studies within each meta-analysis for the effect size indicated by the corresponding meta-analysis, for the three domains of interest. Studies were grouped according to the decile they fell into (0–10%, 11–20%, etc.). This indicated that overall the majority of studies fell into the 0–10% and 11–20% deciles (psychiatric: 42.8%; neurological: 51.0%; somatic: 57.2%), although the distribution clearly showed a second mode of high-powered studies in the 91–100% decile. This pattern is consistent with the distribution previously reported by Button *et al.* [2]. These results are shown in figure 1.

**Figure 2.**
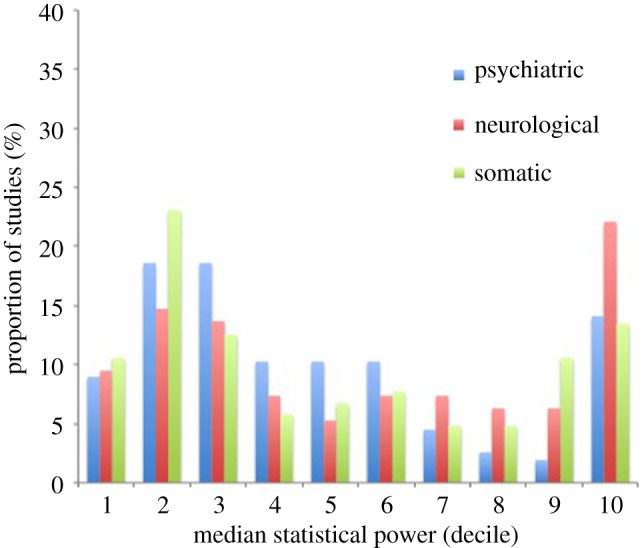
Distribution of statistical power of individual studies (sensitivity analysis). The distribution of the average statistical power of individual studies contributing to meta-analyses across three biomedical domains (psychiatry, neurology and somatic disease) is shown, restricted to meta-analyses indicating a statistically significant pooled effect size estimate only. This indicates a broadly similar bimodal distribution, albeit indicating higher average power overall. This overall pattern again appears to hold across all three domains of interest.

Restricting this analysis to only those meta-analyses that indicated a statistically significant pooled effect size estimate, we found that median statistical power was higher: 38% for somatic diseases, 46% for psychiatric disorders and 50% for neurological diseases (table 2). These results are shown in figure 2, and indicate a broadly similar bimodal distribution to that seen in our primary analysis, albeit with higher average power overall.

**Figure 3.**
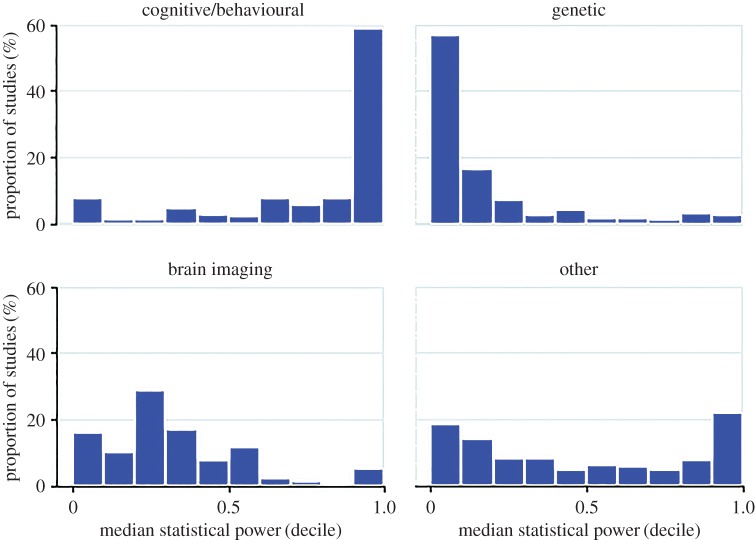
Distribution of statistical power by research methodology. The distribution of the average statistical power of individual studies contributing to meta-analyses across four distinct methodological approaches (cognitive/behavioural, genetic, brain imaging, other) is shown. This clearly shows strong patterning, with cognitive/behavioural studies having high power (majority > 80%), genetic studies very low power (majority < 20%) and brain imaging and other studies occupying an intermediate position. Note that genetic studies comprise candidate gene studies, rather than studies using whole-genome methods (e.g. genome-wide association).

We also plotted average statistical power by research methodology (cognitive/behavioural, genetic, brain imaging, other). This indicated strong patterning by methodology, with cognitive/behavioural studies having high power (median 93%), genetic studies very low power (median 8%), and brain imaging (median 27%) and other studies (median 39%) occupying an intermediate position. These results are shown in figure 3.

### 3.3. Effect size and sample size

**Figure 4.**
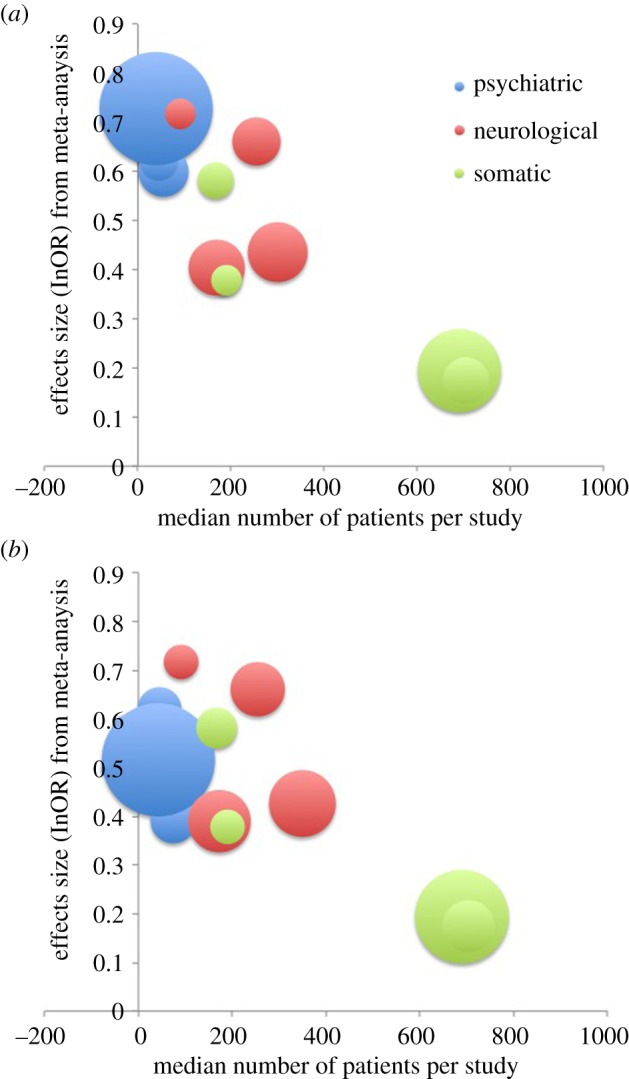
Relationship between sample size and effect size. The relationship between the average number of patients in each study and the effect size indicated by the corresponding meta-analysis across three biomedical domains (psychiatry, neurology and somatic disease) is shown. This clearly indicates a linear relationship between number of patients and effect size. This overall pattern appears to hold across all three domains of interest, and is seen in the full sample of meta-analyses (top panel) and when the subsample of meta-analyses that focus on cognitive or behavioural assessments is excluded (bottom panel). Cluster size is proportional to the number of individual studies in each cluster.

In order to assess the relationship between effect size and sample size we plotted the effect size indicated by the meta-analyses for each disease type against the median number of patients in the studies contributing to those meta-analyses. This indicated a clear linear relationship between effect size and sample size. These results are shown in figure 4.

## 4. Discussion

Our results indicate a striking similarity in average statistical power across three distinct biomedical research domains, and align with similar findings we have previously reported in the neuroscience literature [2], with a median statistical power in the region of 20%. In other words, approximately 50% of studies have power in the 0–10% or 11–20% range, well below the minimum statistical power of 80% that is considered conventional. Low statistical power, therefore, appears to be a problem that is endemic to the biomedical sciences, at least in the specific subject areas captured by our search strategy. This is despite large differences in average sample size—indeed, there is a clear and strong inverse relationship between average sample size in a field and the average effect size observed in that field. We also see evidence, again consistent with our previous work, of a bimodal distribution of the power of individual studies—while the majority of studies appear to have very low power, a minority appears to be very well powered. An important strength of the present study is that we attempted to ensure that the meta-analyses we included, focused on biological, environmental or cognitive correlates of disease, were relatively homogeneous (e.g. by excluding treatment studies). We also sampled a longer period, including meta-analyses published between 2008 and 2012, thereby capturing a much larger number of relevant studies. However, the use of meta-analyses to provide a best estimate of the likely underlying population effect size meant that we were restricted to a subset of the literature within each domain of interest, limiting the extent to which our findings can be generalized to fields where meta-analysis is uncommon.

We conducted a sensitivity analysis excluding meta-analyses where the pooled effect size estimate was non-significant. This was done to reduce the possibility that we were including cases where the true effect size is zero or close to zero. Unsurprisingly average power was higher in this case, although still considerably lower than the 80% that is often conventionally regarded as adequate. On the one hand, including non-significant meta-analyses is likely to reduce our estimate of average power because this will lead to the inclusion of areas where the true effect size is zero or nearly zero. On the other hand, effect size estimates from meta-analyses may be inflated by publication bias against non-significant results. Study quality may also impact on the meta-analytic estimate if there is a systematic relationship between, for example, study sample size and study quality; we used the meta-analytic estimate obtained under a fixed effects model, which places greater weight on larger studies, but larger sample size may be achieved at the expense of measurement precision. An alternative approach would have been to specify what would constitute a biologically or clinically meaningful effect size for each meta-analysis, and exclude those where the pooled effect size estimate was smaller than this. However, this would have been extremely challenging—not only would this have to have been done for each meta-analysis, but it is not clear that such an effect size could be specified in every case. Using a *p*-value threshold instead is somewhat arbitrary but at least practical and transparent. Taken together, we feel that our results are likely to provide a plausible upper and lower bound of the average statistical power within the fields and methodologies sampled. Interestingly, this range aligns well with the results of a recent model that attempted to predict the most rational research strategy, in terms of the proportion of research effort spent on seeking novel results rather than on confirmatory studies, and the amount of research effort per exploratory study [14]. This showed that researchers acting to maximize their ‘fitness’ should spend most of their effort seeking novel results and conducting small studies that have only 10–40% statistical power, even though this means that half of the studies they publish will report erroneous conclusions.

We also observed strong patterning of average statistical power by research methodology. This was not explored in depth by Button *et al.*, although high median power (ranging from 51 to 96%) was observed in five meta-analyses dealing with cognitive/behavioural measures [2]. Consistent with this, studies in the present analysis that used cognitive/behavioural measures showed high average statistical power. By contrast, we previously estimated the median power of structural brain imaging studies to be 8% [2], whereas in this study brain imaging studies had intermediate power around 30%. The reasons for this discrepancy are not clear and warrant further investigation. Those using other measures had average power around or below 50%, while genetic association studies showed very low statistical power. This suggests that the very low power of genetic association studies may contribute to the overall pattern of results we observed across research domains. Importantly, the genetic studies included were exclusively candidate gene studies, and it is now widely acknowledged that results from these studies are not robust, due to a combination of factors such as small effect sizes and small sample sizes [15]. Whole-genome approaches (e.g. genome-wide association) use a combination of very large sample sizes, stringent statistical standards and replication, and the results of these studies are accordingly much more robust [15]. One difficulty in interpreting these results is that certain methodologies (e.g. cognitive/behavioural) were almost exclusively restricted to studies in one research domain (psychiatry). A more comprehensive analysis of the relationship between research methodology and average statistical power is warranted.

The clear linear relationship between effect size and sample size is, on the one hand, to be expected—if researchers conduct power analyses, then sample size should be related to the effect size of the phenomenon being studied. However, this straightforward explanation ignores the fact that average statistical power appears to be low. An alternative explanation is that sample size is governed largely by other factors, such as convention and constraints on resources. If publication bias is operating, whereby only statistically significant findings are published (either because authors choose not to write up non-significant findings, or because journals choose not to publish them, or both), then the filtering effect of a significance threshold (e.g. an *α* level of 0.05) will mean that, for a given average sample size, only effect sizes above a certain magnitude will achieve statistical significance and be published. This would also result in a relationship between observed effect size and sample size. Factors such as analytic flexibility might increase the likelihood of obtaining a statistically significant result in an underpowered study, so that conducting underpowered studies represents a trade-off between the limited resources available and the likelihood of obtaining publishable results. It is worth considering the extent to which incentive structures may shape scientists' behaviour. Publication remains the primary currency of science, and both the quantity and quality of publications by a scientist contribute to their career advancement, success in obtaining grant funding, and so on. Given limited resources, scientists may therefore (consciously or unconsciously) make strategic decisions to maximize the return on those resources, for example, by conducting a relatively large number of small studies (many of which will be publishable, despite those findings potentially being unreliable), while also conducting a smaller number of large, confirmatory studies [14]. However, these factors may differ considerably across countries with different funding systems and incentive structures.

There are some limitations to our analysis that should be considered when interpreting these results. First, and perhaps most importantly, the concept of statistical power is inherently tied to the NHST framework, and the use of a *p*-value threshold (e.g. 0.05) to support statistical inference. This approach, and in particular, a reliance on *p*-value thresholds, has been criticized widely [16], not least because it relies on inferring that a phenomenon exists when statistical evidence passes an arbitrary threshold (typically *p* < 0.05), which in turn implies a demarcation between adequate and inadequate sample size (and power) [17,18]. Nevertheless, the use of *p*-value thresholds to denote whether a result is ‘significant’ or ‘non-significant’ remains a common approach in the biomedical sciences. Second, our method requires the use of information reported in meta-analyses, but meta-analyses may focus only on certain study types. For example, they might focus on the literatures where the answer to the underlying research question remains unclear, in which case our estimates of average power might be underestimates (for example, if that uncertainty is due to small studies being prevalent in that area). On the other hand, they might only include data from high quality studies (as recommended by the Cochrane Collaboration), in which case our estimates might be overestimates. Third, given the very large number of meta-analyses included, we could not check whether any individual studies contributed to more than one meta-analysis. However, this is likely to be uncommon and therefore should not influence our results substantially. Fourth, our method depends critically on the assumption that the effect size indicated by a meta-analysis is the best estimate of any true effect size. We believe that this assumption is valid, not least because accurately estimating effect size is a key purpose of meta-analysis. Relatedly, however, our method assumes that power should always be evaluated at the true effect size. This is invalid if the true effect size is zero (in which case power calculations are meaningless). We attempted to address this by excluding estimates from meta-analyses where the pooled effect size estimate was non-significant, but future studies should also attempt to estimate the power of individual studies to obtain *p* < 0.05 for a minimal biologically or theoretically important effect. Fifth, we relied on the results of fixed effects meta-analysis. This assumes that all studies are drawn from the same underlying population, and attempts to estimate the effect size within that population. Random effects meta-analysis, on the other hand, does not make this assumption, allowing studies to be drawn from multiple underlying populations, and therefore attempting to estimate the range of effect sizes that may exist across these populations. We believe that the assumptions of our analysis align best with those of fixed effects meta-analysis. Sixth, by excluding treatment studies to ensure greater comparability of included studies we have selected for studies where researchers have less control over sample size, or studies that are more exploratory in nature. However, given the range of sample sizes observed in studies within domains, we do not think the first possibility is likely. Also, included studies typically reported inferential statistics, and therefore were (ostensibly at least) hypothesis-testing, rather than exploratory. Seventh, we did not register a protocol for this review prior to conducting the study. However, the largely descriptive nature of our results means, in our opinion, that the scope for analytical flexibility is limited.

There are a number of ways in which research practices might be improved, taking into account the results we report here. First, sample size considerations should feature prominently in the design of studies, using clearer *a priori* estimates of likely effect size that are either based on similar studies in the literature, or considerations of what would be considered clinically or biologically meaningful. The pre-registration of study protocols may help to make these assumptions more transparent, and correctly place an emphasis on statistical power as a pre-study or design consideration [19]. Second, researchers should consider alternative ways of improving statistical power. Here we have focused on sample size, but this will not always be feasible or cost-effective; it may be more appropriate to increase the precision of outcome measures by reducing measurement error (e.g. by collecting more data on each participant). Third, other statistical frameworks or approaches exist that do not rely on the concept of statistical power. While NHST remains common in biomedical science, there are increasing calls to instead use estimation approaches, which focus on estimating the likely effect size and quantifying the precision of this estimate (e.g. using a confidence interval) [20]. This has the particular advantage of not requiring the use of arbitrary thresholds to determine the inferences one should draw from one's data [17]. Another alternative is to use Bayesian methods, which have the particular advantage of providing the probability of a specific hypothesis, given the observed data (rather than the probability of having obtained the observed data, given the null hypothesis). Not only is this potentially more informative, but it also allows evidence for the null hypothesis to be quantified (unlike in NHST, where the interpretation of a non-significant *p*-value can be difficult). In our view, studies should be designed to be informative. Exactly what this means will depend on the approach adopted (NHST, estimation, Bayesian, etc.), and the assumptions that underpin that approach, but certainly within the NHST framework statistical power is a critical design consideration when planning a study. A large body of very small studies providing inconclusive evidence is problematic.

## 5. Conclusion

Our results indicate that low statistical power may be a problem across the biomedical sciences—we find no clear evidence that the average statistical power differs across research into psychiatric, neurological or somatic diseases, but we do find evidence that research methodology may play an important role. This warrants further investigation. The role of current incentive structures in shaping the behaviour of individual scientists in a manner that serves to reduce statistical power also warrants further investigation. If research funders wish to support high-quality research, they should consider funding larger studies, which in turn may entail collaboration across multiple research groups. However, even in the absence of substantial grant funding, researchers can begin to shift research culture towards more powerful research through research training and wider collaboration [21].

## Supplementary Material

Figures S1-S12. Flow charts for inclusion of studies

## Supplementary Material

Table S1. References to included studies

## Supplementary Material

Table S2. Characteristics of included studies

## Data accessibility

The data underlying the analyses reported in this study are included as the electronic supplementary material.
