# Supplementary material for: Low statistical power in biomedical science: a review of three human research domains
Source: R Soc Open Sci. 2017 Feb 1;4(2):160254. doi: 10.1098/rsos.160254 (PMC5367316; doi:10.1098/rsos.160254)
Supplement: Figures S1-S12. Flow charts for inclusion of studies [file rsos160254supp1.doc]

**Figure S1**: Attention Deficit Hyperactivity Disorder

Records identified through PubMed search

(N=118)

Records excluded

(N=75)

Intervention/treatment study (N=27)

Not about ADHD/relevant (N= 12)

Not enough datasets (N=1)

ADHD is the risk factor (N= 4)

Not expressed as d or OR/RR (N=5)

Records screened

(N=118)

Full text articles assessed for eligibility (N= 43)

Full text articles excluded, with reasons

(N=23)

Effect size not reported as d or OR/RR (N=7)

Incomplete data (N=5)

<7 datasets (N=9)

See more recent article about the same topic (N=2)

INCLUDED

Full text articles (N=20)

Meta-analyses (N= 40)

**Figure S2**: Autism

Records identified through PubMed search

(N=71)

Records excluded

(N=35)

Intervention/treatment study (N=27)

Not about Autism/relevant (N= 5)

Autism is the risk factor (N= 3)

Records screened

(N=71)

Full text articles assessed for eligibility (N= 36)

Full text articles excluded, with reasons

(N=23)

Effect size not reported as d or OR/RR (N=13)

Incomplete data (N=3)

<7 datasets (N=7)

INCLUDED

Full text articles (N=13)

Meta-analyses (N= 24)

**Figure S3**: Major Depressive Disorder

Records identified through PubMed search

(N=553)

Records excluded

(N=439)

Intervention/treatment study /diagnosis (N=355)

Not about MDD/relevant (N= 59)

Not enough datasets/incomplete data (N=6)

MDD is the risk factor (N= 14)

Not expressed as d or OR/RR (N=5)

Records screened

(N=553)

Full text articles assessed for eligibility (N= 114)

Full text articles excluded, with reasons

(N=85)

Effect size not reported as d or OR/RR (N=19)

Incomplete data (N=14)

<7 datasets (N=27)

See more recent article about the same topic (N=13)

Not relevant (N=5)

No control group (N=4)

Not a meta-analysis (N=3)

INCLUDED

Full text articles (N=29)

Meta-analyses (N= 54)

**Figure S4**: Schizophrenia

Records identified through PubMed search

(N=454)

Records excluded

(N=256)

Intervention/treatment study /diagnosis (N=171)

Not about SCH/relevant (N= 67)

SCH is the risk factor (N= 17)

Not expressed as d or OR/RR (N=1)

Records screened

(N=454)

Full text articles excluded, with reasons

(N=138)

Effect size not reported as d or OR/RR (N=37)

Incomplete data (N=18)

<7 datasets (N=40)

See more recent article about the same topic (N=19)

Not relevant (N=8)

No control group (N=6)

Not a meta-analysis (N=4)

Qualitative measure (N=4)

Inconsistencies (N=2)

Full text articles assessed for eligibility (N= 198)

INCLUDED

Full text articles (N=60)

Meta-analyses (N= 203)

**Figure S5**: Alzheimer disease

Records identified through PubMed search

(N=197)

Records excluded

(N=80)

Intervention/treatment study /diagnosis (N=67)

Not about AD/relevant (N= 11)

AD is the risk factor (N= 2)

Records screened

(N=197)

Full text articles excluded, with reasons

(N=82)

Effect size not reported as d or OR/RR (N=12)

Incomplete data (N=15)

<7 datasets (N=30)

See more recent article about the same topic (N=7)

Not relevant (N=7)

No control group (N=4)

Not a meta-analysis (N=4)

Qualitative measure (N=1)

Inconsistencies (N=2)

Full text articles assessed for eligibility (N= 117)

INCLUDED

Full text articles (N=35)

Meta-analyses (N= 50)

**Figure S6**: Epilepsy

Records identified through PubMed search

(N=147)

Records excluded

(N=118)

Intervention/treatment study (N=61)

Not about Epilepsy/relevant (N= 51)

Epilepsy is the risk factor (N= 6)

Records screened

(N=147)

Full text articles excluded, with reasons

(N=17)

Effect size not reported as d or OR/RR (N=6)

<7 datasets (N=9)

Not relevant (N=1)

Not a meta-analysis (N=1)

Full text articles assessed for eligibility (N= 29)

INCLUDED

Full text articles (N=12)

Meta-analyses (N= 15)

**Figure S7**: Multiple Sclerosis

Records identified through PubMed search

(N=137)

Records excluded

(N=93)

Intervention/treatment study /diagnosis (N=51)

Not about MS/relevant (N= 35)

MS is the risk factor (N= 7)

Records screened

(N=137)

Full text articles excluded, with reasons

(N=26)

Effect size not reported as d or OR/RR (N=3)

Incomplete data (N=3)

<7 datasets (N=11)

See more recent article about the same topic (N=4)

Not relevant (N=3)

Not a meta-analysis (N=1)

Inconsistencies (N=1)

Full text articles assessed for eligibility (N= 44)

INCLUDED

Full text articles (N=18)

Meta-analyses (N= 37)

**Figure S8**: Parkinson’s Disease

Records identified through PubMed search

(N=139)

Records excluded

(N=65)

Intervention/treatment study (N=43)

Not about PD/relevant (N= 14)

PD is the risk factor (N= 8)

Records screened

(N=139)

Full text articles excluded, with reasons

(N=54)

Effect size not reported as d or OR/RR (N=8)

Incomplete data (N=4)

<7 datasets (N=27)

See more recent article about the same topic (N=10)

Not a meta-analysis (N=5)

Full text articles assessed for eligibility (N= 74)

INCLUDED

Full text articles (N=20)

Meta-analyses (N= 57)

**Figure S9**: Breast Cancer

Records identified through PubMed search

(N=811)

Records excluded

(N=466)

Intervention/treatment study /diagnosis (N=184)

Not about BC/relevant (N= 242)

BC is the risk factor (N= 38)

Not a meta-analysis (N=2)

Records screened

(N=811)

Full text articles assessed for eligibility (N= 345)

Full text articles excluded, with reasons

(N=258)

Effect size not reported as d or OR/RR (N=2)

Incomplete data (N=94)

<7 datasets (N=95)

See more recent article about the same topic (N=57)

Not relevant (N=1)

No control group (N=2)

Not a meta-analysis (N=3)

Inconsistencies (N=4)

INCLUDED

Full text articles (N=87)

Meta-analyses (N= 110)

**Figure S10**: Glaucoma

Records identified through PubMed search

(N=75)

Records excluded

(N=56)

Intervention/treatment study /diagnosis (N=50)

Not about GLAU/relevant (N= 4)

GLAU is the risk factor (N= 2)

Records screened

(N=75)

Full text articles excluded, with reasons

(N=10)

<7 datasets (N=7)

See more recent article about the same topic (N=1)

Not relevant (N=1)

Not a meta-analysis (N=1)

Full text articles assessed for eligibility (N= 19)

INCLUDED

Full text articles (N=9)

Meta-analyses (N= 21)

**Figure S11**: Psoriasis

Records identified through PubMed search

(N=81)

Records excluded

(N=59)

Intervention/treatment study (N=40)

Not about PSO/relevant (N= 14)

PSO is the risk factor (N= 5)

Records screened

(N=81)

Full text articles assessed for eligibility (N= 22)

Full text articles excluded, with reasons

(N=14)

Incomplete data (N=1)

<7 datasets (N=13)

INCLUDED

Full text articles (N=8)

Meta-analyses (N= 15)

**Figure S12**: Rheumatoid Arthritis

Records identified through PubMed search

(N=285)

Records excluded

(N=203)

Intervention/treatment study /diagnosis (N=154)

Not about RA/relevant (N= 34)

RA is the risk factor (N= 15)

Records screened

(N=285)

Full text articles assessed for eligibility (N= 82)

Full text articles excluded, with reasons

(N=60)

Incomplete data (N=3)

<7 datasets (N=31)

See more recent article about the same topic (N=17)

Not relevant (N=9)

INCLUDED

Full text articles (N=22)

Meta-analyses (N= 34)
